# Effects of a liquefied petroleum gas stove intervention on pollutant exposure and adult cardiopulmonary outcomes (CHAP): study protocol for a randomized controlled trial

**DOI:** 10.1186/s13063-017-2179-x

**Published:** 2017-11-03

**Authors:** Magdalena Fandiño-Del-Rio, Dina Goodman, Josiah L. Kephart, Catherine H. Miele, Kendra N. Williams, Mitra Moazzami, Elizabeth C. Fung, Kirsten Koehler, Victor G. Davila-Roman, Kathryn A. Lee, Saachi Nangia, Steven A. Harvey, Kyle Steenland, Gustavo F. Gonzales, William Checkley, Cardiopulmonary outcomes and Household Air Pollution trial (CHAP) Trial Investigators

**Affiliations:** 1 Division of Pulmonary and Critical Care, School of Medicine, Johns Hopkins University, 1830 E. Monument St. Room 555, Baltimore, MD 21205 USA; 2 Department of Environmental Health and Engineering, Bloomberg School of Public Health, Johns Hopkins University, Baltimore, MD USA; 3 Department of International Health, Bloomberg School of Public Health, Johns Hopkins University, Baltimore, MD USA; 4 Cardiovascular Division, Department of Medicine, Washington University, St. Louis, MO USA; 5 Department of Environmental Health, Rollins School of Public Health, Emory University, Atlanta, GA USA; 6 Department of Biological and Physiological Sciences, Faculty of Sciences and Philosophy, Universidad Peruana Cayetano Heredia, Lima, Peru

**Keywords:** Cookstove, LPG, Indoor air pollution, Household air pollution, Personal exposure, Biomass fuel, Cardiopulmonary outcomes, Behavior change, Exclusive adoption

## Abstract

**Background:**

Biomass fuel smoke is a leading risk factor for the burden of disease worldwide. International campaigns are promoting the widespread adoption of liquefied petroleum gas (LPG) in resource-limited settings. However, it is unclear if the introduction and use of LPG stoves, in settings where biomass fuels are used daily, reduces pollution concentration exposure, improves health outcomes, or how cultural and social barriers influence the exclusive adoption of LPG stoves.

**Methods:**

We will conduct a randomized controlled, field intervention trial of LPG stoves and fuel distribution in rural Puno, Peru, in which we will enroll 180 female participants aged 25–64 years and follow them for 2 years. After enrollment, we will collect information on sociodemographic characteristics, household characteristics, and cooking practices. During the first year of the study, LPG stoves and fuel tanks will be delivered to the homes of 90 intervention participants. During the second year, participants in the intervention arm will keep their LPG stoves, but the gas supply will stop. Control participants will receive LPG stoves and vouchers to obtain free fuel from distributors at the beginning of the second year, but gas will not be delivered. Starting at baseline, we will collect longitudinal measurements of respiratory symptoms, pulmonary function, blood pressure, endothelial function, carotid artery intima-media thickness, 24-h dietary recalls, exhaled carbon monoxide, quality-of-life indicators, and stove-use behaviors. Environmental exposure assessments will occur six times over the 2-year follow-up period, consisting of 48-h personal exposure and kitchen concentration measurements of fine particulate matter and carbon monoxide, and 48-h kitchen concentrations of nitrogen dioxide for a subset of 100 participants.

**Discussion:**

Findings from this study will allow us to better understand behavioral patterns, environmental exposures, and cardiovascular and pulmonary outcomes resulting from the adoption of LPG stoves. If this trial indicates that LPG stoves are a feasible and effective way to reduce household air pollution and improve health, it will provide important information to support widespread adoption of LPG fuel as a strategy to reduce the global burden of disease.

**Trial registration:**

ClinicalTrials.gov, ID: NCT02994680, Cardiopulmonary Outcomes and Household Air Pollution (CHAP) Trial. Registered on 28 November 2016.

**Electronic supplementary material:**

The online version of this article (doi:10.1186/s13063-017-2179-x) contains supplementary material, which is available to authorized users.

## Background

Household air pollution (HAP), caused by the combustion of biomass fuels (typically wood, dung, and agricultural crop waste), is a leading contributor to the global burden of disease and the largest environmental risk factor for preventable disease [1–3]. HAP from biomass fuel smoke was estimated to be responsible for 4.3 million deaths in 2012 [4]. It has been associated with chronic bronchitis, chronic obstructive pulmonary disease (COPD) [5–13], lung cancer [14, 15], childhood pneumonia [16], acute lower respiratory infections [17, 18], cardiovascular events [19, 20], and low birthweight [21, 22]. Globally, nearly three billion individuals, 40% of all households and 90% of rural households use biomass fuels as their primary source of domestic energy [2]. Burning of biomass fuels for cooking is most pervasive in low- and middle-income countries (LMIC). Women and children have the highest risks of exposure to biomass fuel smoke, accounting for 60% of premature deaths from HAP in 2012 [23]. HAP may also have important effects on both cardiovascular and pulmonary health.

Cardiovascular disease (CVD) is the leading cause of morbidity and mortality worldwide [24]. More than 80% of CVD deaths occur in LMICs, where age-adjusted rates of cardiovascular-related deaths can be up to five times higher than in high-income countries [25]. By 2030, CVD will be responsible for 75% of deaths worldwide, more than 24 million deaths, and will account for more deaths in LMICs than infectious diseases, maternal and perinatal conditions, and nutritional disorders combined [24–26]. Targeting preventable causes of CVD in LMICs, such as HAP, is imperative. To date, no field intervention trial has sufficiently demonstrated that reducing HAP can decrease CVD morbidity, leaving policy-makers reluctant to implement cleaner stove programs as a strategy to address CVD risk.

### Cardiovascular disease related to biomass fuel smoke exposure

There is a growing understanding that pulmonary inflammation caused by HAP affects cardiovascular health [27]. However, less is known about the mechanism by which HAP leads to CVD or the true burden of biomass fuel smoke exposure on CVD risk [1, 27]. Cross-sectional and longitudinal studies have found strong associations between ambient air pollution, measured by particulate matter (PM) levels, and an increased risk of cardiovascular-related death [27–32]. Chronic exposure to biomass fuels has been associated with a thicker carotid artery intima-media complex, higher prevalence of atherosclerotic plaques, and higher blood pressure [33]. PM less than 2.5 μm in diameter (PM_2.5_), including ultrafine particles, are small enough to deposit in alveoli, initiate inflammatory cascades, and enter the pulmonary circulation [34]. The Women’s Health Initiative Observational Study followed more than 65,000 women from urban areas for 6 years and found that an increase of 10 μg/m^3^ in ambient PM_2.5_ was associated with a 24% increased risk of cardiovascular events and a 76% increased risk of cardiovascular-related deaths [20]. However, little is known about the response curve of HAP on cardiovascular outcomes [35]. Investigators in Guatemala found that when biomass fuel smoke-related PM exposure was reduced by 50% via an improved biomass-burning stove, a reduction in blood pressure within a few months was found in a sample of women [36]. PM is believed to affect the circulatory system through increased blood pressure, inflammation, propagation of coagulation, and increased blood viscosity [27, 37–39]. Particularly, endothelial dysfunction and progression of atherosclerosis are believed to be an integral link between PM exposure and worse cardiovascular health [30]. There is clear evidence that ambient air pollution causes both acute [30–32, 34] and chronic endothelial dysfunction [40, 41], but the relationship with HAP has not been fully explored in intervention trials.

### Respiratory disease related to biomass fuel smoke exposure

The adverse health effects of HAP on respiratory health, and specifically its contribution to COPD, have been well documented [42–47]. A previous study in rural Peru found that 55% of COPD prevalence can be attributed to daily exposure to biomass fuel smoke [48]. Among women, biomass fuel smoke exposure was associated with a doubling in the odds of COPD [48] and chronic bronchitis [49], similar to that described in other studies [46]. Interventions in rural Mexico revealed a consistent relationship, with improved wood stoves being associated with reductions in respiratory symptoms [13, 50]. However, there is still an important gap in our understanding of how biomass fuel smoke exposure affects lung function decline [47]. Previous findings suggest a log-linear relationship between HAP and lower respiratory infections, i.e., a steep slope for PM_2.5_ levels below 100 μg/m^3^ and a less pronounced slope thereafter [35, 51]. This implies that very low levels of exposure need to be achieved to observe health benefits.

### Previous research on improved stoves

Improved cookstoves were introduced as a cost-effective strategy to reduce HAP [16, 44, 52–55] through more efficient combustion of biomass fuels and ventilation. Most modifications include closed combustion chambers and chimneys [47, 56]. Although improved cookstoves have achieved important reductions in HAP, both personal exposure and kitchen concentrations of environmental pollutants remain several-fold higher than World Health Organization (WHO)-recommended levels [57], and the effect of these achieved reductions on health outcomes has not always been evident [52]. For example, a randomized field trial of improved biomass fuel stoves in Guatemala did not lower physician-diagnosed pneumonia (primary outcome) in the intention-to-treat analysis (RR = 0.84, 95% CI 0.63–1.13) between children in the intervention and control arms [16]. Similarly, a recent, large-scale cluster-randomized trial in Malawi did not reduce childhood pneumonia with an improved biomass-burning stove intervention [58]. The lack of health effects for both of these trials is likely attributed to the continued use of polluting stoves and an inability of the intervention to achieve reductions in personal exposure below the WHO intermediate targets (35 μg/m^3^) of household air quality. Prior exposure-response analyses have found that the greatest risk reductions occur with much lower exposure levels that are unlikely to be achieved with improved biomass-burning stoves [2, 16, 35]. For example, in RESPIRE, the exposure-response analyses found that an expected reduction of 50% in personal carbon monoxide (CO) concentrations was associated with a lower incidence of physician-diagnosed pneumonia (RR = 0.82, 95% CI 0.70–0.98) but the intention to treat analysis did not show significant results. The authors attribute the difference in results between the intention-to-treat and exposure-response analyses to less exposure misclassification in the latter, i.e., the intention-to-treat analysis does not account for subject-specific exposures to HAP. Moreover, larger expected reductions may lower the risk of physician-diagnosed pneumonia even further. Thus, recent intervention efforts are shifting towards stoves that use cleaner fuels [53, 54, 59–62].

Current research has demonstrated that liquefied petroleum gas (LPG) fuels can significantly reduce HAP when compared to biomass fuels. However, there have been few studies that have reported on the relationship between direct measures of HAP and health outcomes using LPG stoves as an intervention to replace biomass stoves [54]. Studies in Guatemala and Bangladesh demonstrated PM reductions with LPG stove use that ranged from 45 to 90%, although these studies were cross-sectional and enrolled a small number of participants [53, 54, 61].

Additionally, few studies have linked LPG stove use to health outcomes [8, 59, 62–66], and even fewer have incorporated longitudinal follow-up of HAP and health [59]. Using retrospective, longitudinal data, a study in China concluded that women cooking with cleaner fuels, including LPG, were more likely to report better health and had a lower probability of chronic and acute diseases when compared to women cooking with biomass fuels [59]. A cross-sectional study in a rural village near Mexico City found that women cooking with biomass fuels had more respiratory symptoms and higher kitchen PM_10_ concentrations, compared to those cooking with LPG [67]. A cross-sectional study of 760 women in rural India found that 43% of those using biomass fuels had abnormal peak expiratory flow (PEF) readings when compared to 23% of those using LPG [62]. Cataracts have also been associated with the use of biomass fuels for cooking [2], and this also appears to be reduced with LPG. A case-control study along the Nepal-India border found that women using traditional cookstoves were 90% more likely to develop cataracts than women using a combination of biogas, LPG, and kerosene (OR = 1.90, 95% CI 1.00–3.61) [63]. This study, however, did not consider the effects of LPG stove use alone, and included kerosene [63], which has been classified by the WHO as a dangerous fuel source [57]. Overall, there is a lack of longitudinal research studies examining the health effects of switching from biomass fuel to LPG for cooking.

A major barrier to assessing the effect of LPG fuel use on HAP and health is stove stacking, in which families continue using their traditional biomass stoves after receiving an LPG stove. Since just 1 h per week of traditional stove use raises PM_2.5_ concentrations above WHO guidelines, even a small amount of stove stacking can negate many of the benefits of clean cooking technologies [68]. Previous research has shown that people who use LPG stoves like the speed of cooking, the cleanliness, and ease of use, but that the cost of fuel is frequently a barrier to adoption [69]. Additional barriers to exclusive adoption of LPG stoves include fears that LPG stoves are unsafe, lack of knowledge about their proper operation, a desire for more burners, and changes in the taste of food cooked on LPG stoves [69]. However, previous research on the adoption of LPG stoves has focused on situations where LPG must be purchased. To date, there is no research on factors that might hinder or facilitate adoption once cost is removed as an obstacle [69]. Thus, it is necessary to gain an in-depth understanding of factors that motivate families to adopt clean fuels and facilitate the exclusive use necessary to achieve the potential health benefits.

Previous research has rarely examined other consequences of stove interventions such as changes in diet and quality of life. Anderman et al. observed that households provided with methane biogas stoves had more diverse diets (measured by number of food groups) than households using a firewood stove [60]. Nonetheless, little is known about how LPG stoves might affect dietary patterns. One of the few studies that has looked at quality of life and biomass burning found that respiratory quality of life improved after installation of ventilated cookstoves in rural Bolivia [70]. To date, no intervention has assessed how a gas stove intervention affects quality-of-life outcomes.

There is a lack of studies with sufficient sample size and longitudinal follow-up to determine the health effects of LPG fuel use when compared to biomass fuel use. Additionally, few studies have collected high-quality environmental measurements of both kitchen and personal air quality. Furthermore, many studies have struggled to reduce HAP concentrations due to stove stacking. This study aims to address these gaps by conducting a longitudinal assessment of clinical outcomes, and personal exposure and kitchen HAP concentrations over 2 years, while explicitly investigating and incorporating behavior change into study activities. Understanding the factors influencing LPG adoption will enable us to better motivate exclusive LPG adoption and better quantify the potential impact of LPG stoves on exposure to HAP and health. Because no field intervention trial of LPG fuel use has convincingly demonstrated that a reduction of HAP decreases morbidity or mortality, policy-makers have been reluctant to invest in its more widespread adoption. This trial could provide evidence needed to justify investment in LPG as an effective public health intervention.

## Methods

### Study objectives

**Fig. 1:**
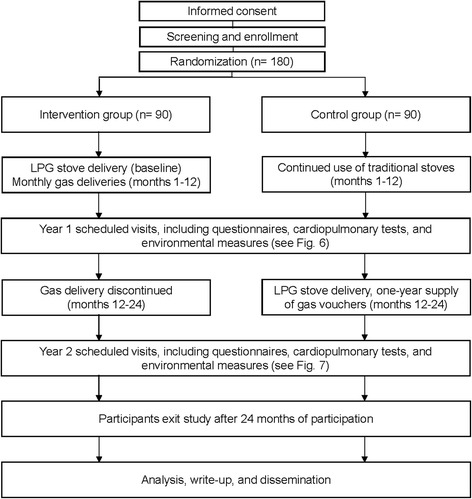
Expected study enrollment and timeline diagram

This study is a randomized, field intervention trial testing the efficacy of LPG stove use and fuel distribution, compared to traditional, open-fire stove use, as a strategy to reduce HAP and improve cardiopulmonary outcomes in the rural, high-altitude setting of Puno, Peru. LPG stove use will be monitored and compared to standard cooking practices to determine the relative effect of LPG adoption on HAP concentrations and subsequent improvements in cardiopulmonary outcomes over a 1-year period. As a secondary objective, in the second year of follow-up, we will measure intervention effectiveness by characterizing the sustainability of LPG among participants in the intervention arm and initial adoption of LPG among those in the control arm (Fig. 1).

### Study setting and population

**Fig. 2:**
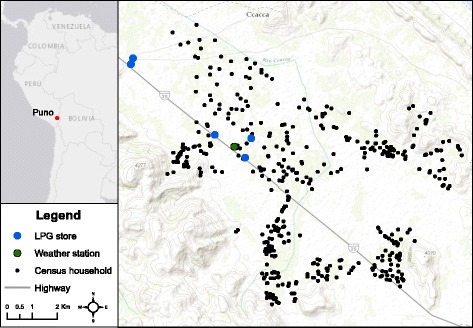
Location of all households in the study population and liquefied petroleum gas fuel purchase sites

**Fig. 3:**
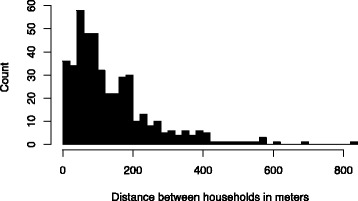
Barplot of nearest-neighbor distances between households in study area. Median distance to nearest house between households in the study area is 101 m with an interquartile range of 56 to 189 m

The trial will take place in rural areas surrounding the city of Puno in southeastern Peru (Fig. 2), on the shore of Lake Titicaca at 3825 m above sea level. Puno is the capital and largest city of the Puno Province which, in 2007, had a population of 230,000 inhabitants [71]. Study participants live in rural communities in the province of Puno, where biomass-burning, open-fire stoves are used for cooking [72]. Median distance to nearest house between all households in the study area is 101 m with an interquartile range of 56 to 189 m (Fig. 3). Median nearest distance to the highway for households in the study area is 1701 m with an interquartile range of 893 to 3103 m. Only 4% of households are within 100 m from the road.

We seek to enroll 180 women, 90 in both the intervention and control arms. One woman per household will be enrolled. To be eligible for the trial, women must be aged 25–64 years, be the primary cook, be a full-time resident in their current location for at least 6 months, be capable of understanding study procedures, providing informed consent, and respond to questionnaires, use biomass fuels daily for cooking, and have a cooking area separate from their sleeping area. The latter criterion was added to exclude households that use biomass fuel stoves to heat their living space. Women will be excluded from participating if they plan to move from the area within 1 year, have hypertension (taking antihypertension medications, or systolic blood pressure ≥ 140 mmHg or diastolic pressure ≥ 90 mmHg) or a diagnosis of COPD (post-bronchodilator FEV_1_/FVC below the lower limit of normal of a reference population), smoke cigarettes daily, are pregnant or have plans to become pregnant in the next year, and have active pulmonary tuberculosis or are taking antituberculosis medications for pulmonary tuberculosis. Women in these communities speak Aymara (local language) and Spanish. Field staff members who will collect data from participants are native Spanish speakers, and at least one member of the field team who speaks Aymara will attend each visit.

### Intervention

**Fig. 4:**
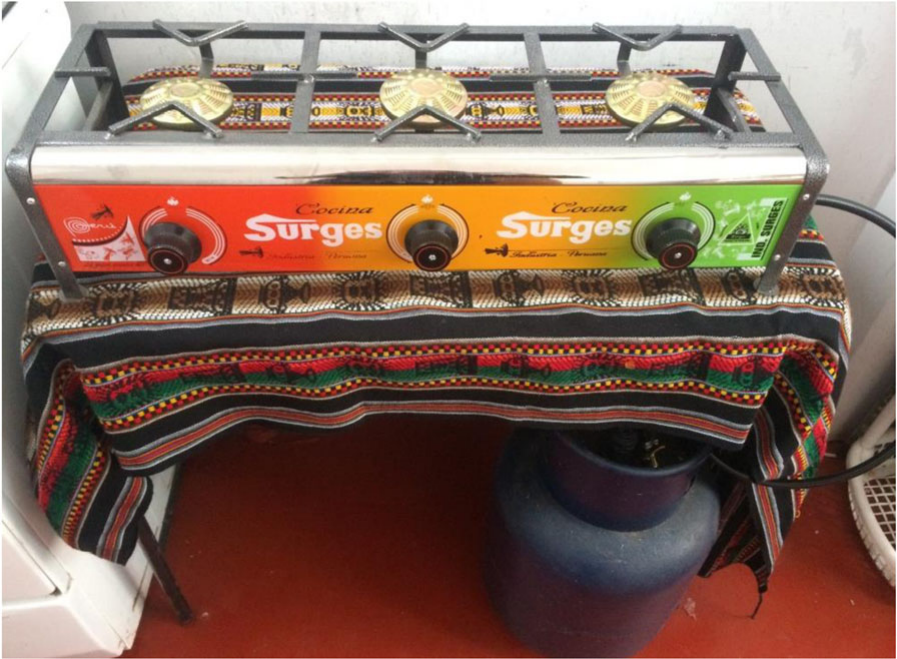
Three-burner liquefied petroleum gas stove that will be used for the intervention

Participants will be provided with an LPG stove, with three burners, that connects to an external gas tank (Fig. 4). Formative research showed that three-burner stoves were preferred by the community over the commonly available two-burner stoves. Stoves will be purchased from a local manufacturer (Industrias SURGES, Juliaca, Peru). Current open-fire stoves will not be replaced or destroyed by study personnel. LPG fuel will be locally purchased and delivered to intervention participants’ homes for the first year of the study. Control participants will receive vouchers to pick free fuel from distributors for the second year of the study. Before receiving an LPG stove, all intervention participants will attend a community meeting where they will observe a cooking demonstration and receive behavioral messages based on formative research to promote exclusive LPG stove use. As part of the cooking demonstration, participants will receive safety information and training on how to correctly operate and maintain the LPG stove. Correct and exclusive use of the LPG stove will be reinforced during the approximately bi-monthly gas delivery visits to the households.

### Study design

**Fig. 5:**
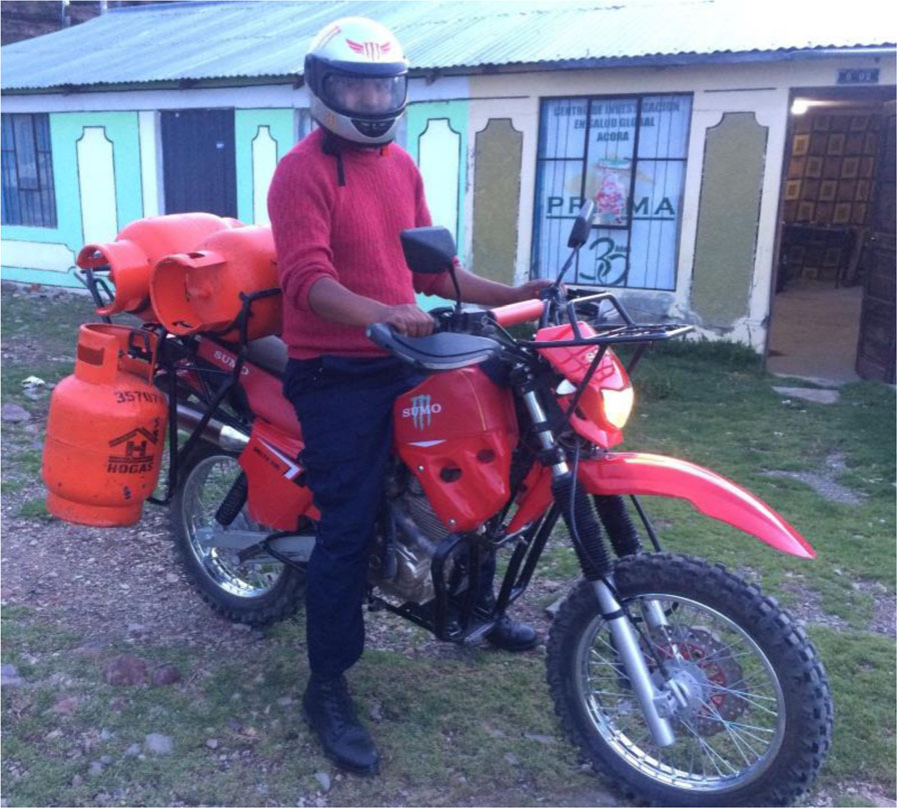
Delivery system through motorcycle for liquefied petroleum gas tanks

Each participant who is enrolled in the study will receive a set of baseline pre-intervention assessments and multiple follow-up assessments over 24 months. The 90 participants assigned to the intervention arm will receive a free LPG stove and free fuel delivery to their homes approximately twice monthly during the first year to ensure a steady supply (Fig. 5). Rate of gas use will be tracked by a field staff member. Participants will be assigned to the intervention or control arms with a 1:1 ratio using random permuted block sizes of 2, 4, and 6 [73]. The randomization schedule was created in R [74] by an investigator who will not be involved in the recruitment or baseline interviews of participants. Study-arm allocation will be masked from field staff in envelopes until baseline measurements are complete. A field staff member will then open the envelopes.

The study will be conducted over 3 years, with staggered enrollment over the first year. Our field team will start recruitment by meeting with the leaders of each community to explain the study, obtain approval to approach community residents for participation, and schedule community meetings before enrollment. Our team will use previously collected census data to identify potentially eligible households. At the beginning of the study, we will randomly determine the order in which communities and subsectors within communities will be visited. Each month for the first year, we will randomly select households within subsectors from the census list to obtain informed consent and assess eligibility. We aim to enroll 15 households each month.

A nurse will be present during enrollment and clinical evaluations to thoroughly explain the trial, the procedures, and answer questions. We will obtain verbal informed consent (i.e., waiver of documentation of consent) and we will leave a written copy of the Consent Form signed by study staff with each participant. Any serious adverse events will be identified by the study team who will generate electronic reports within 24 h after the event. Participants would be directed towards the nearest emergency health facility for appropriate medical care. However, since this is a low-risk study, we do not expect any serious adverse events.

**Fig. 6:**
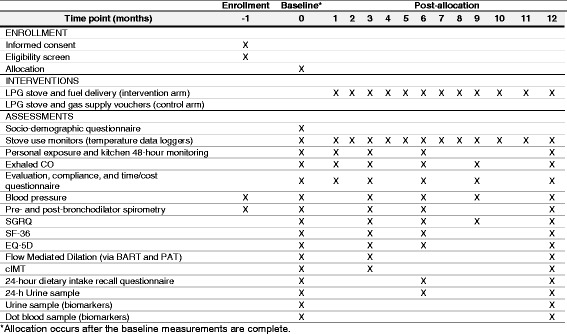
Standard Protocol Items: Recommendations for Interventional Trials (SPIRIT) Figure data collection schedule for the first year of follow-up

After obtaining informed consent, all participants will be asked to answer questions about their sociodemographics, medical history and current clinical symptoms. Personal exposure and kitchen 48-h PM_2.5_ and CO concentrations will be measured for each participant at baseline and longitudinally during the first year. We will monitor nitrogen dioxide (NO_2_) in a subset of 100 participants (50 in both the intervention and control arms). Stove use will be assessed by continuously monitoring the temperature of LPG and traditional stoves. Health outcomes will be measured at baseline and repeatedly at different intervals in the first year (Fig. 6). Primary outcomes include respiratory symptoms, spirometry, blood pressure, endothelial function and carotid intima-media thickness, and quality of life. Secondary outcomes include dietary salt intake and other macronutrients, inflammatory markers of HAP in urine and blood, and exhaled CO.

**Fig. 7:**
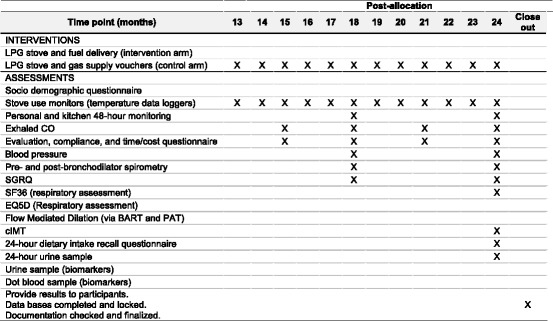
Standard Protocol Items: Recommendations for Interventional Trials (SPIRIT) Figure data collection schedule for the second year of follow-up

At the end of the 12-month intervention period, control-arm participants will receive a free LPG stove and vouchers to cover a 1-year supply of fuel. Participants will be followed for a second year to determine patterns of sustained use (intervention arm) or initial adoption (control arm), and their effects on HAP and health outcomes (Fig. 7).

### Study organization

The study will be implemented in partnership by Asociación Benéfica PRISMA (A.B. PRISMA) in Lima, Peru; Universidad Peruana Cayetano Heredia in Lima, Peru; and Johns Hopkins University, Baltimore, MD, USA. Technical support will be provided by experts from Emory University in Atlanta, GA, USA and Washington University in Saint Louis, MO, USA. This study was approved by the Institutional Review Boards of A.B. PRISMA and Universidad Peruana Cayetano Heredia in Lima, Peru, and the Johns Hopkins Bloomberg School of Public Health in Baltimore, MD, USA.

### Sample size

We based sample size calculations on preliminary data obtained by our group from prior studies [75] and feasibility intervention trials on the association between HAP and blood pressure, PEF and St. George’s Respiratory Questionnaire (SGRQ) (Table 1). To measure a 5-mmHg (SD = 10) lowering of systolic blood pressure (SBP), a 20-L/min/m^2^ (SD = 40) improvement in height-adjusted PEF, and a 10-point (SD = 20) higher SGRQ result between intervention and control arms with 80 to 90% power and 95% confidence, we need to enroll at least 63 to 85 participants in each arm. These estimations are consistent with previously observed differences in health outcomes [75–77]. Thus, we aim to enroll 180 women (90 in each arm) to account for potential dropouts and loss to follow-up.

**Table 1:** Number of participants needed in each arm for primary outcomes

Outcome of interest	Effect size	Standard deviation	Sample size (power 90%)	Sample size (power 80%)
Blood pressure	5 mmHg	10	85	63
Peak expiratory flow	20 L/min/m^2^	40	85	63
St. George’s Respiratory Questionnaire (score)	10	20	85	63

### Stove adoption and social behavioral components

After enrollment, we will invite intervention arm participants to a group meeting that will include a cooking demonstration with local ingredients and recipes, developed based on formative research. This session will include information about using an LPG stove safely and present motivational messaging to encourage participants to use LPG exclusively.

We will conduct longitudinal, qualitative, in-depth interviews to explore factors related to LPG adoption. Three rounds of interviews will be conducted. First, we will interview 10 participants who use their LPG stove for ≥ 80% of cooking events and 10 participants who use their LPG stove for < 80% of cooking events at 1–3 months post intervention. We will explore differences in barriers, motivators, practices, and preferences between these two types of LPG stove users to identify strategies for promoting exclusive adoption. The same 20 households will then be asked to participate in a second interview, after owning their LPG stove and receiving free fuel for 10–12 months, to determine how their perceptions and stove-use practices may have changed over time. Finally, we will return to these 20 households for a third interview, at 15–17 months post intervention, to understand factors influencing sustained use or abandonment of LPG stoves when fuel is no longer provided for free. Additional interviews may be conducted to ensure adequate representation of exclusive and non-exclusive LPG stove users.

We will also conduct quarterly behavioral questionnaires to collect information on observed and self-reported use of all household stoves, reasons for stove use, time spent cooking and collecting biomass fuels, and fuel expenditures. In intervention households, we will also collect information on the likes and dislikes of the LPG stove, stove maintenance and repair behaviors, and overall opinions of LPG stoves. These visits will be used to reinforce exclusive use of LPG through behavioral messaging and address any problems or concerns with the gas stove. We will continuously measure stove use during the 2 years on both LPG and traditional stoves using the Digit-TL temperature monitor (LabJack Corporation, Lakewood, CO, USA).

### Environmental assessment

**Fig. 8:**
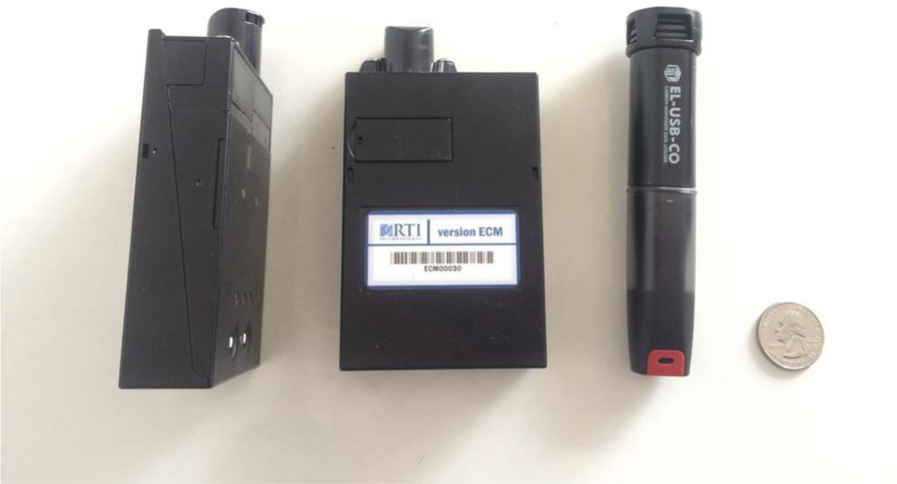
Portable particulate matter and carbon monoxide sampling devices

**Fig. 9:**
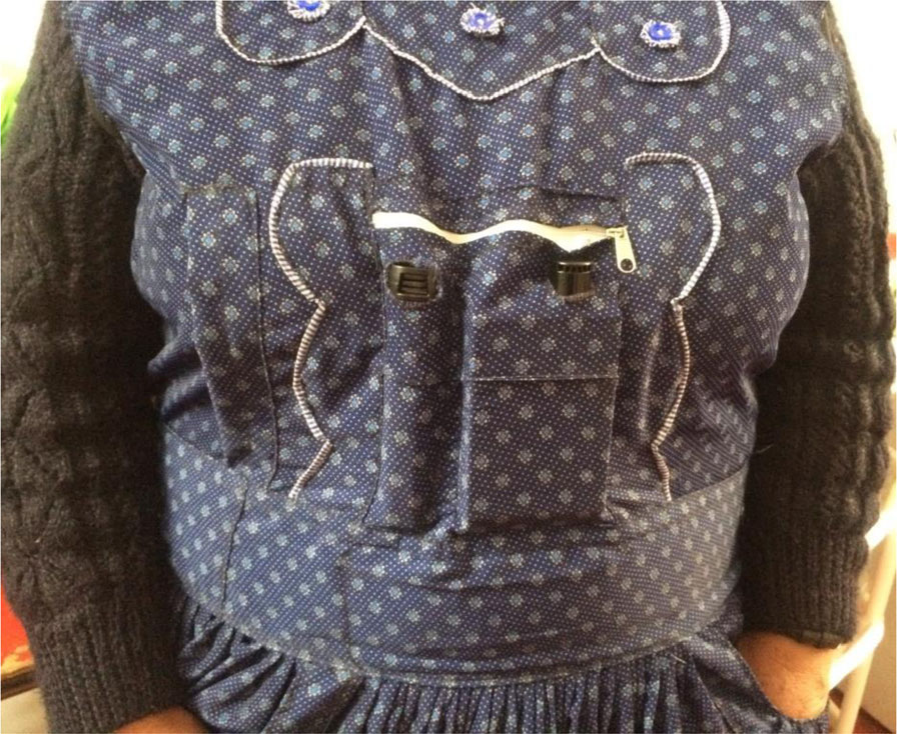
Placement of devices in an apron pocket to monitor personal exposure of particulate matter of less than 2.5 μm in aerodynamic diameter (PM_2.5_) and carbon monoxide (CO)

We will measure 48-h personal exposure and kitchen concentrations of PM_2.5_ with the ECM Monitor (RTI Inc., Research Triangle Park, NC, USA) and CO with the EL-USB-CO data logger (Lascar Electronics, Erie, PA, USA) at each environmental assessment visit. Both the ECM and EL-USB-CO are light-weight monitors and can be easily worn by participants without disrupting daily activities (Fig. 8). The ECM has a light-scattering laser for real-time assessment of PM_2.5_, and a 0.3-L/min pump that will be continuously on for 48 h to gravimetrically collect PM_2.5_ in a 15-mm diameter filter. We will calibrate the ECM pumps daily with a TSI 4100 flowmeter (TSI Incorporated, 500 Cardigan Road, Shoreview, MN, USA). We will use 15-mm Teflon filters with a 2-μm membrane (Measurement Technology Laboratories LLC, Minneapolis, MN, USA). All filters will be pre- and post-weighed in a humidity- and temperature-controlled room using a XP2U microbalance (Mettler Toledo, Columbus, OH, USA) located in the Department of Environmental Health and Engineering of the Bloomberg School of Public Health at Johns Hopkins University. We will conduct direct readings of PM_2.5_ every 3 out of 10 s and time-weight average these values into 1-min intervals, and conduct direct readings of CO in 1-min intervals. We will ask participants to wear the ECM and CO monitors near the breathing zone in the pocket of an apron provided to them. The aprons, which are similar to those commonly used by women in our setting, were selected as the most feasible and acceptable method for personal exposure sampling during formative research (Fig. 9). Participants will be encouraged to wear the aprons during awake hours and co-locate them near their beds when sleeping.

We will measure kitchen concentrations of PM_2.5_ and CO using the same instruments described above. Monitors will be placed approximately 1 m from the combustion zone, at 1.5 m of height from the floor, and at least 1 m away from doors and windows, when possible. We will also measure personal exposure and kitchen concentrations of NO_2_ in a subsample of households using the Aeroqual 500 series monitors (Aeroqual Limited, Auckland, New Zealand). Passive NO_2_ samplers from Ogawa (Ogawa USA, Pompano Beach, FL, USA) will measure personal exposure to NO_2_ in a subsample of 20 women. Passive NO_2_ samples will be analyzed using standard methods [78].

Kitchen samples of PM_2.5_ will include 10% blanks and 10% duplicates, and all reported concentrations will be blank-corrected. Kitchen CO samples will include 10% duplicates. Calibration checks will be performed every 3 months for the CO monitors using a chamber to test the devices with clean air and two, different, known CO concentrations. Staff will also record general characteristics of the kitchen including wall materials, and the presence of windows and doors.

Ambient particulate matter pollution (pDR-1000, Thermo Fisher Scientific, Wharton, MA, USA) and meteorology (Vantage VUE, Davis Instruments, Hayward, CA, USA) will be monitored in a central location during the study period.

### Pulmonary health outcomes

We will measure respiratory symptoms with the SGRQ. The SGRQ is a standardized, self-completed questionnaire for measuring impaired health and perceived wellbeing in individuals with chronic airway disease [78]. This questionnaire is easy to use, gives a comparative measurement of respiratory health between populations, and can be used to quantify changes in respiratory health following interventions [79]. It has been translated and validated for use in Spanish.

We will measure exhaled CO using the Micro CO Meter (Micro Direct, Lewiston, ME, USA) as an additional measure of compliance. We will take two exhaled CO measurements and average them to obtain the final value. The Micro CO Meter will be calibrated monthly with a 20-ppm CO concentration gas cylinder connected directly to the device.

We will use the EasyOn PC handheld spirometer (ndd Medical Technologies Inc., Zurich, Switzerland) to assess lung function [42, 43]. The EasyOn PC has an ultrasonic flow reader that is not affected by air density and is suitable for use at high altitudes. We will check spirometer calibration weekly using a 3-L syringe (Hans Rudolph Inc., Kansas City, MO, USA). If a spirometer reads more than 3.5% above or below 3 L [80], it will not be used in the field and will be repaired or replaced. All team members involved in administering tests will be trained to comply with standard guidelines [80]. Regular supervision and feedback will take place via a centralized quality-control program [81]. We will record forced vital capacity (FVC), forced expiratory volume in 1 s (FEV_1_), peak expiratory flow (PEF), and store individual flow-volume curves for quality-control assessment and further analysis. Using a salbutamol inhaler approved for use by the Peruvian General Directorate of Medication, Drugs, and Supplies, we will administer two puffs (100 mcg/puff) via a spacer and repeat spirometry after 15 min for reversibility testing, i.e., an improvement of > 12% or > 0.2 L in baseline forced expiratory volumes.

We will measure oxyhemoglobin saturation with a Rad 5v pulse oximeter (Masimo, Irvine, CA, USA). We will take two oxyhemoglobin saturation measurements and average them to obtain the final value.

### Cardiovascular health outcomes

SBP and diastolic blood pressure (DBP) will be measured in triplicate in 5-min intervals and in the sitting position using an automatic blood pressure monitor OMRON HEM-780 (Omron, Tokyo, Japan). At enrollment, we will determine the arm with the highest SBP and will use that arm thereafter for all measurements. We will average the second and third values to determine final SBP and DBP.

cIMT is a noninvasive, surrogate marker of atherosclerosis and has also been shown to predict future cardiovascular events [82, 83]. Increased thickness of the carotid artery is associated with increased risk for stroke, myocardial infarction, and other adverse cardiovascular outcomes as previously shown by our group and others [33, 83, 84]. We will follow standard guidelines for cIMT assessment [85–88]. A high-frequency portable ultrasound (M-Turbo, Sonosite Inc., Bothell, WA, USA) with a linear transducer (6–13 MHz) will be used to visualize the carotid vessel. Special attention will be paid to obtain vessel interface and carotid plaques, wall thickness of the distal common carotid artery, and Doppler velocity. The cardiac cycle will be tracked with an electrocardiogram (EKG) monitor built into the ultrasound [89] for cIMT assessment. We will evaluate subjects in the morning after an overnight fast. cIMT images will be saved in DICOM format on a local server, and then uploaded to a secure Cloud server (Ultralinq, New York, NY, USA) for transfer to the USA.

Endothelial function will be measured using brachial artery reactivity testing (BART) [90] and peripheral arterial tonometry (PAT) [91], which will be administered simultaneously. BART and PAT are non-invasive methods for assessing endothelial function and are associated with future CVD risk [92–94]. A higher BART score, indicating increased flow-mediated dilation (FMD) in the brachial artery, is associated with better cardiovascular health. A systematic review of 23 studies and 14,753 participants found that a 1% higher FMD was associated with an 8% lower risk of a future CVD event [94]. We will use portable ultrasound (M-Turbo, Sonosite, Bothell, WA, USA) with a high-frequency linear probe (6–13 MHz) to visualize the brachial artery in longitudinal view following standard guidelines [95]. We will measure the diameter of the vessel at end diastole before and after distal arm ischemia. We will use a blood pressure cuff immediately distal to the antecubital fossa and inflate the cuff to 240 mmHg to obtain sufficient occlusion for distal arm ischemia. We will maintain cuff inflation for 5 min and measure post-ischemia vessel diameter at 30, 60, 90, 120, and 150 s after cuff deflation and calculate the percentage change in diameter from baseline to each post-ischemia measurement. Time of dilation related to cardiac cycle will be tracked with an EKG monitor built into the ultrasound [89]. BART images will be saved in DICOM format on a local server, and then uploaded to a secure Cloud server (Ultralinq, New York, NY, USA) for transfer to the USA.

Image analysis for BART and cIMT will be done at Washington University. All studies will be analyzed by two sonographers who will be blinded. The primary reader will evaluate all tests from the study, while the secondary reader (expert) will evaluate 10% of the tests at random for quality control. Intra- and inter-observer intra-class correlation coefficients at the Washington University Core Laboratory for BART and CIMT measurements are greater than 0.91.

An EndoPAT (Itamar, Caesarea, Israel) will be used for PAT. Finger pulse-wave amplitude will be recorded with the EndoPAT probe placed on the index finger of the same arm for BART. A second EndoPAT probe will be placed on the contralateral index finger. A reactive hyperemia index will be recorded and normalized. The EndoPAT device documents the percentage change between pre- and post-ischemia tests. EndoPAT data will be saved in a local server for FTP transfer. Data analysis will be done at Johns Hopkins.

### Anthropometry and nutrition outcomes

Each participant will have weight and height (standing and sitting) measured at baseline and at 12 months post intervention using a standardized protocol. Dietary intake will be assessed through 24-h recalls. Participants will describe what they consumed both individually and in shared meals. Whenever possible, food will be weighed using a Henkel Max 5000-g d-1G scale (HV Digital Eirl, Lima, Peru). Otherwise, participants will be asked to identify portion sizes visually using a standardized book developed by A.B. PRISMA [96]. This book contains to-scale pictures of common food items, plate-ware and corresponding weight in grams. Salt intake will be assessed in a subset of 100 participants with a 24-h urine test. Participants will be provided with a receptacle to urinate for a 24-h period. The samples will be mixed and aliquoted before shipment to a clinical laboratory (Medlab, Arequipa, Peru) for analysis. The sodium measured in urine will be used to validate salt intake recorded in the 24-h recalls.

### Quality-of-life outcomes

We will measure quality of life with the RAND 36-Item Short Form Health Survey (SF-36) [97] and the EuroQol five dimensions questionnaire (EQ5D) [98]. We will use these quality-of-life indicators to calculate differences in quality-adjusted life years between the intervention and control arms. The validated Spanish versions of both the SF-36 and the E5QD are being used in this study.

### Urine and blood markers

Women will be provided with a 500-mL urine collection cup and instructed to urinate briefly to waste before collecting the remainder of the urine void in the cup. The time of urine collection and time of previous urine void (if known) will be recorded and the total volume collected will be estimated and recorded. The urine will be transferred in its entirety to 3-oz Qorpak bottles and labeled. The labeled bottles will be secured in freezer boxes and will be stored in a cooler with ice packs until they can be transported to a − 20 °C freezer.

Blood will be collected as dried blood spots (DBS). A finger from the non-dominant hand will be swabbed with a sterile alcohol wipe. The sterile lancet will be used to puncture the skin and the initial drop of blood will be wiped away with an alcohol swab. Blood from the finger will be allowed to drip onto five standard spots on a Guthrie DBS card. The finger will be squeezed until a large drop of blood appears on the finger and the drop will be quickly applied to an unfilled spot on the card. Each spot will contain approximately 100 μL blood so the total blood collection will be less than 1 mL. Cards will be labeled and dried at room temperature (20–25 °C) on drying rack for 10 h without the use of external heat or fan. Dried cards will be placed in individually labeled ziptop bags into which a desiccant pouch and a humidity indicator card will be placed. Urine and DBS samples will be blinded to assignment before biomarker analysis at Emory University (Table 2).

**Table 2:** Panel of biomarkers

Biomarker	Purpose	Matrix	Method
Intercellular adhesion molecule 1 (ICAM-1)	Endothelial marker of cardiovascular function	DBS	Immunoassay
Vascular cellular adhesion molecule 1 (VCAM-1)	Endothelial marker of cardiovascular function	DBS	Immunoassay
Endothelin-1	Endothelial marker of cardiovascular function	DBS	Immunoassay
E-selectin	Endothelial marker of cardiovascular function	DBS	Immunoassay
C-reactive protein (CRP)	Inflammation marker	DBS	Immunoassay
Interleukin-6 (IL-6)	Inflammation marker	DBS	Immunoassay
von Willebrand factor antigen (vWF)^a^	Blood coagulation protein	DBS	Immunoassay
Hemoglobin A1C (HbA1C)	Marker of glycemic control	DBS	Spectrophotometry
Hemoglobin (Hb)	Clinical biomarker	DBS	Spectrophotometry
Lipids^a^	Clinical biomarkers	DBS	Immunoassay
P53 tumor-associated antigen antibodies (p53 TAA antibodies)^a^	Lung and other cancer biomarker	DBS	Array assay
F2-isoprostanes	Inflammation markers	DBS	Immunoassay
Clara cell protein (CC16)	Lung insult marker	DBS	Immunoassay
8OH-deoxyguanosine (8OHdG)	Oxidative stress marker	Urine	LC-MS/MS
Myeloperoxidase (MPO)^a^	Oxidative stress marker	DBS	Immunoassay
Cytochrome P450 (Cyp450)^a^	Enzyme induction	DBS	Immunoassay
4-OH cotinine	Short term tobacco smoke biomarker	Urine	LC-MS/MS
4-(methylnitrosamino)-1-(3-pyridyl)-1-butanol (NNAL)^a^	Tobacco smoke biomarker		
Polycyclic aromatic hydrocarbons (1-OH pyrene and 1-, and 2-naphthols)	Carcinogen exposure biomarker	Urine	GC-MS/MS
Volatile organic chemicals (mercapturate metabolites)^a^	Carcinogen exposure biomarker	Urine	LC-MS/MS

^a^Subject to sample volume availability

Most clinical biomarkers will be analyzed in duplicate through immunoassay methods with the MesoScale MSD Mutiplexer Clinical Analyzer. We will perform one reagent blank per run, three quality-control materials per run, and eight calibration samples per plate. For mass spectrometry-based methods we will use Agilent 7000 Triple quadrupole MS/MS with a gas chromatograph and electron impact and chemical ionization capabilities. We will perform calibrations, reagent blanks, and matrix blanks every 25 samples.

### Biostatistical analysis

We will conduct intention-to-treat analyses of cardiovascular and pulmonary health endpoints (primary outcomes) during the first year of the intervention. We will calculate 48-h personal exposure and kitchen PM_2.5_ and CO concentrations, and compare differences in these concentrations between treatment arms. We will use linear mixed-effects models to examine the effect of the intervention on subject-specific trajectories of cardiovascular (SBP, DBP, metrics of FMD, and cIMT) and pulmonary (SGRQ score, PEF, and FEV_1_) endpoints. As a sensitivity analysis, we will evaluate exposure reduction and clinical outcome relationships. Specifically, we will ignore random allocation and instead we will examine if there is an exposure-response relationship between reduction in pollutant concentrations and change in clinical outcomes. For this analysis, we will use linear mixed-effects models to measure the subject-specific changes in clinical outcomes in relation to reductions of personal exposure and kitchen concentrations. Since this type of analysis breaks randomization, we will adjust for any potential confounders.

Stove-use monitoring data will be summarized to determine the number of cooking events and cooking duration per day for each participant in LPG and traditional stoves. Stove-use monitoring will also be useful in exposure-response analyses described above.

Secondary analyses will focus on effectiveness outcomes during the second year. Specifically, we plan to calculate the cumulative incidence of abandonment of LPG stoves and percentage use of LPG stoves in the intervention arm, adoption of LPG stoves in the control arm, and factors affecting these statistics. When participants decide to drop out, field staff will complete a questionnaire to document potential biases related to missing data. We will incorporate recommended methods from the academic literature and sensitivity analysis to properly account for missing data [99].

Qualitative data will be analyzed inductively, coding transcripts with emerging themes using ATLAS.ti (ATLAS.ti, Berlin, Germany). Information from coded quotes will be synthesized and compared within and across themes.

### Data management and quality assurance

Questionnaires and other field forms will be collected on tablets using the Research Electronic Data Capture software (REDCap, Vanderbilt University Medical Center, Nashville, TN, USA) [100]. Images collected from BART, PAT, and cIMT will be uploaded to a secure Cloud server (Ultralinq Health Care Solutions, New York, NY, USA). Twenty-four-hour food recalls will be collected on paper forms, combined with nutrition facts from official Peruvian Ministry of Health documents [101, 102], and entered into a database. Qualitative interviews will be audio-recorded, transcribed by local field staff into Spanish, and then translated into English. Data processing and storage will be centralized at a server in Puno, Peru. Personal information will be maintained confidential before, during, and after the trial.

The study team will be in constant contact with the study site team with weekly scheduled meetings and additional communication as needed for quality control. All data that is collected will be de-identified and uploaded onto Cloud servers for real-time access. As study procedures are completed, the study team will have real-time oversight of all activities.

Publications on the results and analysis of the primary outcomes and main objectives will be prioritized over those on secondary outcomes and objectives. We will follow the Consolidated Standards of Reporting Trials (CONSORT) 2010 guidelines when reporting the main results of the trial [103]. Paper topics and authorship will be discussed with the respective team leaders of each component and the principal investigator. In accordance with NIH data-sharing policy, we will submit our de-identified, limited dataset to the National Heart, Lung and Blood Institute Biologic Specimen and Data Repository after publication [104].

## Discussion

LPG stoves have been recently proposed as a strategy to achieve WHO air-quality guidelines in LMICs, but there are limited studies, much less studies with repeated measurements, which measure the effects of LPG stove use on HAP and adult cardiopulmonary outcomes. Similarly, there is little information on potential outcomes if sustained LPG stove adoption is achieved. To address these gaps, we plan to conduct an LPG field intervention trial with extensive behavioral support and repeated HAP and health outcome assessments to understand how LPG adoption could reduce disease burden in LMICs.

We will measure stove use, personal exposure and kitchen pollutant concentrations, and cardiopulmonary outcomes repeatedly during 2 years. We will monitor biomarkers of exposure and nutritional changes. Longitudinal measurements over 2 years will allow us to identify short-term health benefits of the intervention. Randomization will provide confidence in the comparability between intervention and control arms. If this trial demonstrates that LPG stoves reduce HAP and improve health, it would provide critical evidence that shifting from biomass to LPG fuel stoves is an effective way to reduce the burden of cardiopulmonary-related illnesses and death.

One of the challenges in cookstove interventions is the difficulty of ensuring exclusive LPG stove use. Study participants may continue to use their traditional open-fire stoves for various reasons. First, these open-fire stoves will not be removed from the home and participants will be free to use either stove. To address this challenge, we will incorporate regular visits to reinforce the exclusive use of LPG stoves. Second, participants may not like the taste of food or may believe that they cannot cook traditional dishes like in an open-fire stove. Therefore, we have incorporated cooking demonstrations to show participants that it is possible to prepare traditional dishes with an LPG stove that taste similar to meals cooked on an open-fire stove. Third, participants may have safety concerns that may deter the use of LPG stoves, but this will also be addressed through education during our field visits. Fourth, achieving a consistent supply of LPG fuel to our study participants may be challenging. To address this potential concern, we have incorporated strategies to monitor LPG use and deliver fuel tanks based on usage rates.

The second year of our study will provide valuable information on continued use and factors related to abandonment or adoption of LPG stoves. These results will inform scale-up and implementation of future LPG programs. An additional challenge is the lack of outdoor air-quality measurements in our study area to control for external sources of pollutants that could affect HAP, including neighbors with biomass-burning stoves. To characterize ambient air pollution in our study area, air quality will be monitored in a central location. However, given the large distances between houses in our rural setting, cross-contamination between households is less likely.

While we hypothesize that LPG stoves will reduce HAP and improve cardiopulmonary outcomes, there are other sources of energy, such as electricity, which are likely to produce even greater benefits through a more consistent supply and lower emissions. However, electric stoves are not currently appropriate for many communities around the world because of the lack of access to electricity. Moreover, LPG has become more widely available with a supply infrastructure to meet demands in many countries. In Peru, the communities in our study area have experience with LPG stoves and there are existing government initiatives to make LPG more affordable.

### Trial status

The trial has been registered at www.clinicaltrials.gov (NCT02994680). Trial has not started enrollment at the time of manuscript submission. Enrollment is expected to begin on 18 January 2017. This protocol paper for this trial complies with the SPIRIT (Standard Protocol Items: Recommendations for Interventional Trials) [105] Checklist and the World Health Organization Trial Registration Items [106] (Figs. 6 and 7, Additional files 1 and 2).

## Additional files

Additional file 1: SPIRIT Checklist. (PDF 682 kb)

Additional file 2: World Health Organization Trial Registration Data Set Items. (PDF 45 kb)

## Abbreviations

A.B. PRISMA: Asociación Benéfica PRISMA
BART: Brachial artery reactivity testing
cIMT: Carotid intima-media thickness
CO: Carbon monoxide
COPD: Chronic obstructive pulmonary disease
CVD: Cardiovascular disease
DBP: Diastolic blood pressure
DBS: Dried blood spots
EKG: Electrocardiogram
EQ5D: EuroQol five dimensions questionnaire
FEV_1_: Forced expiratory volume at 1 s
FMD: Flow-mediated dilation
FVC: Forced vital capacity
HAP: Household air pollution
IRB: Institutional Review Board
LMIC: Low- and middle-income countries
LPG: Liquefied petroleum gas
NIH: National Institute of Health
NO_2_: Nitrogen dioxide
PAT: Peripheral arterial tonometry
PEF: Peak expiratory flow
PM: Particulate matter
PM_2.5_: PM less than 2.5 μm in aerodynamic diameter
SBP: Systolic blood pressure
SF-36: 36-Item Short Form Health Survey
SGRQ: St. George’s Respiratory Questionnaire
SPIRIT: Standard Protocol Items: Recommendations for Interventional Trials
WHO: World Health Organization
