# Supplementary material for: Effects of a liquefied petroleum gas stove intervention on pollutant exposure and adult cardiopulmonary outcomes (CHAP): study protocol for a randomized controlled trial
Source: Trials. 2017 Nov 3;18:518. doi: 10.1186/s13063-017-2179-x (PMC5670728; doi:10.1186/s13063-017-2179-x)
Supplement: Supplementary file 2 — World Health Organization Trial Registration Data Set Items. (PDF 45 kb) [file 13063_2017_2179_MOESM2_ESM.pdf]

World Health Organization Trial Registration Data Set Items for CHAP

**Data Category**	**Information**
Primary Registry and Trial Identifying Number	Cardiopulmonary Outcomes and Household Air Pollution Trial (CHAP): NCT02994680.
Date of Registration in Primary Registry	Registered 28 November 2016.
Secondary Identifying Numbers	Johns Hopkins School of Public Health Institutional Review Board: IRB00007128.<br>A.B. PRISMA Ethical Institutional Committee: CE2402.16<br>Universidad Peruana Cayetano Heredia Institutional Review Board: SIDISI 66780
Source(s) of Monetary or Material Support	National Institute of Environmental Health Sciences, United States National Institutes of Health (1U2RTW010114-01), and the Global Alliance for Clean Cookstoves of the United Nations Foundation (UNF 16-80) and the Johns Hopkins Center for Global Health
Primary Sponsor	Fogarty International Center, United States National Institutes of Health
Secondary Sponsors	Global Alliance for Clean Cookstoves of the United Nations Foundation
Contact for Public Queries	William Checkley, MD, PhD<br>Division of Pulmonary and Critical Care<br>School of Medicine, Johns Hopkins University<br>1800 Orleans Ave Suite 9121<br>Telephone: (443) 287-8741<br>Baltimore, MD 21287, USA<br>E-mail: wcheckl1@jhmi.edu
Contact for Scientific Queries	William Checkley, MD, PhD<br>Division of Pulmonary and Critical Care<br>School of Medicine, Johns Hopkins University<br>1800 Orleans Ave Suite 9121<br>Telephone: (443) 287-8741<br>Baltimore, MD 21287, USA<br>E-mail: wcheckl1@jhmi.edu
Public Title	Cardiopulmonary Outcomes and Household Air Pollution Trial
Scientific Title	Effects of a liquefied petroleum gas stove intervention on pollutant exposure and adult cardiopulmonary outcomes: study protocol for the CHAP randomized controlled trial
Countries of Recruitment	Peru
Health Condition(s) or Problem(s) Studied	Cardiovascular disease and respiratory disorders associated with household air pollution

Intervention	Participants in the intervention arm will receive liquefied petroleum gas (LPG) stove and fuel delivered to their homes during the first year of the study. Participants in the control arm will receive LPG stoves and vouchers to obtain free fuel from distributors for the second year of the study (gas will not be delivered). We seek to enroll 180 women, 90 in both the intervention and control arms. One woman per household will be enrolled.<br><br>Participants will be provided with an LPG stove with three burners that connects to an external gas tank. LPG fuel will be locally purchased and delivered to intervention participant's homes for the first year of the study. Control participants will receive vouchers to pick free fuel from distributors for the second year of the study.<br><br>Before receiving an LPG stove, all intervention participants will attend a community meeting where they will observe a cooking demonstration and receive behavioral messages based on formative research to promote exclusive LPG stove use. As part of the cooking demonstration, participants will receive safety information and training on how to correctly operate and maintain the LPG stove. Correct and exclusive use of the LPG stove will be reinforced during the approximately bi-monthly gas delivery visits to the households.
Key Inclusion and Exclusion Criteria	Inclusion Criteria: Women must be aged 25-64 years, be the primary cook, be a full-time resident in their current location for ≥6 months, be capable of understanding study procedures, providing informed consent, and responding to questionnaires, use biomass fuels daily for cooking; and have a cooking area separate from their sleeping area.<br><br>Exclusion criteria: Women must not be planning to move from the area within one year, have hypertension (taking anti-hypertension medications or systolic blood pressure ≥ 140 mmHg or diastolic pressure ≥ 90 mmHg) or a diagnosis of COPD (post-bronchodilator FEV_1/FVC below the lower limit of normal of a reference population), smoking cigarettes daily, pregnant or planning to be pregnant in the next year, or active pulmonary tuberculosis or taking anti-tuberculosis medications for pulmonary tuberculosis.
Study Type	CHAP is an interventional, randomized, and parallel trial.<br>Masking of some analysts: the sonographers that will be assessing endothelial function, and the urine and dried blood spots analysts will be blinded. Study arm allocation will be masked from field staff in envelopes until baseline measurements are complete.<br><br>The purpose of this field intervention trial is to test the efficacy of LPG stove use and fuel distribution, compared to traditional, open-fire stove use as a strategy to reduce HAP and improve cardiopulmonary outcomes in the rural, high altitude setting of Puno, Peru. LPG stove use will be monitored and compared to standard cooking practices to determine the relative effect of LPG adoption on HAP concentrations and subsequent improvements in cardiopulmonary outcomes over a one-year period.<br>As a secondary objective, in the second-year of follow-up, we will measure intervention effectiveness by characterizing the sustainability of LPG among participants in the intervention arm and initial adoption of LPG among those in the control arm.

Date of First Enrollment	Enrollment started January 18, 2017
Target Sample Size	180 women (90 in both the intervention and control arms)
Recruitment Status	Recruiting: participants are currently being recruited and enrolled. The study will be conducted over three years, with staggered enrollment over the first year.
Primary Outcome(s)	Outcome Name: Blood Pressure.<br>Metric/method of measurement: millimeters of mercury (mmHg), using an automatic blood pressure monitor.<br>Time point: At baseline and after 3, 6, 9, 12, 18, and 24 months of follow-up.<br><br>Outcome Name: Peak Expiratory Flow.<br>Metric/method of measurement: liters per minute per meters squared (L/min/m2) measured by pre- and post-bronchodilator with a spirometer.<br>Time point: At baseline and after 3, 6, 9, 12, 18, and 24 months of follow-up.<br><br>Outcome Name: Respiratory quality of life.<br>Metric/method of measurement: St. George's Respiratory Questionnaire with a score.<br>Time point: At baseline and after 3, 6, 9, 12, 18, and 24 months of follow-up.
Key Secondary Outcomes	Outcome Name: Endothelial function and carotid intima media thickness.<br>Metric/method of measurement: A high-frequency portable ultrasound; Metric: millimeters and centimeters per second for Doppler amplitude.<br>Time point: At baseline and after 3, 6, 9, and 12 months of follow-up.<br><br>Outcome Name: Exhaled carbon monoxide.<br>Metric/method of measurement: parts per million (ppm) using a Micro CO Meter.<br>Time point: At baseline and after 1, 3, 6, 9, 12, 15, 18, 21, and 24 months of follow-up.<br><br>Outcome Name: Personal exposure and kitchen 48-hour fine particulate matter, carbon monoxide and nitrogen dioxide (NO_2) concentrations.<br>Metric/method of measurement: concentrations micrograms per cubic meter and parts per million ($\mu g/m^3$, ppm) using a gravimetric and real time the ECM Monitor, with the EL-USB-CO data logger and active and passive NO_2 samplers.<br>Time point: At baseline and after 1, 3, 6, 12, 18, and 24 months of follow-up.<br><br>Outcome Name: Inflammatory markers.<br>Metric/method of measurement: Concentration in blood and urine.<br>Time point: Urine sample: At baseline and after 3, and 12 months of follow-up. Dry blood spot sample: At baseline and after and 12 months of follow-up.<br><br>Outcome Name: Dietary salt intake.<br>Metric/method of measurement: 24-hour dietary recall and 24-hour urine sample. Metric: milligrams/24-hours.<br>Time point: At baseline and after 6, 12, and 24 months of follow-up.
